# Adolescent Sexual Behavior in Rural Central India: Challenges and Interventions

**DOI:** 10.7759/cureus.49761

**Published:** 2023-11-30

**Authors:** Rahul U Ramteke, Jagadish G Makade, Gulshan R Bandre

**Affiliations:** 1 Forensic Medicine, Shri Shankaracharya Institute of Medical Sciences, Bhilai, IND; 2 Community Medicine, Datta Meghe Medical College, Datta Meghe Institute of Higher Education and Research (DU), Nagpur, IND; 3 Microbiology, Jawaharlal Nehru Medical College, Datta Meghe Institute of Higher Education and Research (DU), Wardha, IND

**Keywords:** reproductive healthcare, sex education, rural central india, sexual behavior, adolescent

## Abstract

Adolescence is a crucial life stage marked by significant physical, psychological, and societal changes. With India projected to have the highest population of teenagers by 2025, understanding adolescent sexual behavior in rural central India is essential due to its unique social and cultural contexts. This article reviews existing literature to explore the prevalence, risk factors, and consequences of teenage sexual behavior in rural central India. It highlights the challenges posed by societal taboos, limited access to sexual health information and services, and the impact of poverty on adolescents' sexual behavior and health outcomes. To address these issues, comprehensive sex education, improved access to contraception and reproductive health services, and efforts to overcome cultural and societal norms are crucial. The article discusses the initiatives undertaken by the government and non-governmental organizations (NGOs) to tackle adolescent sexual behavior and emphasizes the need for a multifaceted approach that addresses systemic issues while empowering adolescents. It concludes by suggesting future research directions and policy recommendations aimed at promoting safe sexual behavior among rural adolescents in central India. This article will discuss the complexity of adolescent sexual behavior in rural central India, its origins, and the challenges faced by medical decision-makers.

## Introduction and background

Adolescence is a critical life stage characterized by profound changes in the body, mind, and society. India has the 253 million adolescent population which is largest in the world [1]. By 2025, India is predicted to have the highest population of teenagers in the whole world due to the country's strong adolescent population growth. Adolescent development must include sexual behavior since it affects their health, happiness, and opportunities for the future [2]. It's critical to comprehend how teenagers behave sexually in rural parts of India since they have different social and cultural contexts from metropolitan areas [3]. Sexual behavior is a crucial component of this evolution during adolescence, a period of tremendous physical and psychological change. During adolescence, hormonal changes manifest in the development of secondary sexual characteristics, such as breast growth in females and facial hair in males. Concurrently, adolescents experience a myriad of physical transformations, like growth spurts, alongside psychological variations, including identity exploration and heightened emotional intensity. These hormonal, physical, and psychological changes collectively define the dynamic nature of the adolescent age group. In contrast to metropolitan regions, rural central India has a diverse societal backdrop, and sexual behavior is frequently surrounded by taboos and misunderstandings. With a focus on the prevalence, risk factors, and effects of sexual behavior in this community, this review of the literature attempts to investigate the knowledge currently available on teenage sexual behavior in rural central India [4]. However, sexuality is frequently shrouded in taboos, myths, and shame in many cultures and countries, including rural central India. As a result, it might be difficult for teens to obtain credible sexual health information and resources [5]. According to studies, there are several issues with sex behavior that teenagers in rural central India must deal with, such as early sexual initiation, a lack of access to contraception and reproductive healthcare, and insufficient sex education. In addition, sexuality-related societal and cultural norms may discourage youth from seeking support or coming out with their worries. An increased risk of unintended pregnancies, sexually transmitted diseases (STDs), and unsafe abortion can result from this [6].

It is crucial to offer thorough sex education that addresses subjects like reproductive health, contraception, and safe sexual practices to address these issues. This instruction needs to be given in a setting that fosters open communication between instructors, parents, and students' peers. The availability of contraception-safe abortion services and access to other reproductive health treatments should also be increased [7]. The cultural and societal issues contributing to the shame and taboo around sexuality in rural central India must also be addressed. Working with community leaders, religious leaders, and other influential individuals may be necessary to spread knowledge of the value of sexual health and encourage acceptance of open discourse about sexuality [8]. Adolescents in rural central India confront difficulties because of socioeconomic reasons, including poverty and illiteracy. Adolescents from low-income households may not have access to sexual health-related information, materials, and healthcare services, rendering them more susceptible to unfavorable results. Additionally, in poorer areas, sexuality-related cultural and societal norms may be considerably more prevalent, which further complicates the problem [9]. Adopting a multifaceted strategy that addresses both human and systemic issues is crucial to overcoming these obstacles. This may entail expanding educational chances, giving young people job options, and advancing gender equality. Additionally, it can entail collaborating with local authorities and medical professionals to raise knowledge of and access to reproductive health services and develop welcoming conditions that promote candid conversation about sexuality [8].

In this article, we discuss various factors contributing to adolescent sexual behavior, cultural and social norms related to teenage sexuality, access to sexual health education and services, impact of poverty, consequences, and initiatives taken by government and non-governmental organizations (NGOs) to address adolescent sexual behavior and its challenges and barriers to implementing effective interventions.

## Review

Prevalence of adolescent sexual behavior

Studies have reported varying prevalence rates of adolescent sexual behavior in rural central India. According to a study by Maaan et al. [10], approximately 15% of adolescents in rural central India reported sexual experience. Another study reported a higher prevalence of sexual activity among adolescents, with over 30% of respondents reporting sexual experience [11]. The prevalence of early sexual debut (before 18 years of age) was also reported to be high in rural central India, with a report that over 50% of adolescents had engaged in sexual activity before the age of 18 [12].

Factors contributing to adolescent sexual behavior in the region

Teenage refers to the subset within adolescence, encompassing individuals aged 10-19 [13]. Teenage sexual behavior is influenced by many variables in rural central India. The lack of knowledge and instruction regarding sexual health is one of the main causes. In many places in India, notably rural ones, the topic of sex education is still taboo. Parents are hesitant to bring it up with their children because it is frequently not taught in schools. Due to their lack of knowledge, adolescents are more likely to be misinterpreted and have sexual beliefs that result in unsafe sexual behavior [14]. Teenager's adherence to social and cultural standards about sexual behavior is another problem. Premarital sex is often seen as unacceptable and stigmatized in rural regions of central India. Many young people engage in extramarital relationships due to a lack of importance placed on marriage. This results in increased risk for STDs, unintended pregnancies, and other health issues as a result of limited sexual health education [10,15]. Another important element is the influence of poverty on the sexual behavior of adolescents. Many young people in central rural India lack basic resources such as food, housing, and healthcare due to poverty in the area. Poverty also affects their education, and many people have to leave school to support their families. Because of their lack of financial security and education, they are more likely to engage in risky sexual acts [16].

Cultural and social norms related to adolescent sexuality in rural central India

Cultural and social norms significantly affect adolescent sexual behavior in rural central India. Teenagers find it difficult to discuss sex freely due to the stigma around it. They are concerned about being assessed and criticized by their family and society in general. Premarital sex is usually regarded as evil, and young people who indulge in it are labeled as promiscuous [17]. Gender norms can have a substantial impact on adolescent sexual behavior. Gender roles are still strongly entrenched in rural central India, with men and women doing different chores and taking on different duties. Girls are expected to be meek and submissive, whereas guys are expected to be rough and macho. Because of the gender imbalance, males are typically more dominant and females are more submissive in sexual encounters [18].

Access to sexual health education and services in rural central India

There are minimal resources for sexual health education and care in rural central India. Many youths do not have access to information on safe sexual practices, contraception, or sexual health. They are more vulnerable to STDs and unexpected pregnancies as a result of their lack of education. Even if they want to seek help, they usually face several hurdles, such as a lack of transportation, poor infrastructure, and social shame [10]. Due to a lack of equipment and financing in many health centers, rural people also have limited access to healthcare. There aren't enough personnel with the required training to assist teens with counseling and care, and healthcare professionals are usually overburdened. Teenagers who do not have access to healthcare services are more likely to have sexual health issues [19].

The impact of poverty on adolescent sexual behavior and health outcomes

Poverty has a significant impact on adolescent sexual behavior and health outcomes in rural central India. Their education is impacted by poverty, which leaves them open to sex-related myths and misinformation [20]. Additionally, it limits their ability to access medical care, making it challenging for them to get assistance when they do. Their mental health and self-esteem are affected by poverty, which increases their propensity for unsafe sexual behavior [21]. Teenagers from households with poor incomes are more likely to have unprotected sexual activity, which raises their risk of STDs and unintended pregnancies. They struggle to participate in safe sex since their access to contraception is limited due to poverty. They are at a higher risk of health issues because of their lack of knowledge and financial stability, which makes it harder for them to make educated decisions regarding their sexual health [22].

Consequences

Sexual behavior in adolescents can have serious physical and psychological repercussions. The physical effects of teenage sexual behavior include unwanted pregnancies, STDs, and unsafe abortions [13]. Teenagers who participate in sexual activity run the psychological risk of developing depression, anxiety, and other mental health conditions. In rural central India, where premarital sex is frequently frowned upon, adolescents who engage in sexual behavior run the danger of social shame and rejection [23].

Government and NGO initiatives to address adolescent sexual behavior in the region

Several programs have been started by the Indian government and NGOs to address teenage sexual behavior in rural central India. The Adolescent Reproductive and Sexual Health (ARSH) program is one of the many efforts the government has started to encourage sexual health education. The ARSH package of services includes promotive, preventive, curative, and referral services. These services aim to promote good health, prevent diseases, provide treatment when necessary, and refer patients to specialized care if needed [24]. Adolescents are intended to get complete healthcare services through the ARSH programmer, including counseling, education, and medical treatment [25]. A large part of advancing sexual health education in rural regions has been performed by NGOs. They collaborate with locals to promote sexual health awareness and give them access to medical treatment. To enhance the standard of care given to adolescents, NGOs teach medical personnel [26].

Challenges and barriers to implementing effective interventions in rural central India

The implementation of efficient treatments to deal with adolescent sexual behavior in rural central India is hampered by many difficulties and obstacles. The lack of finance and resources is one of the major issues. Many rural health centers lack the tools and resources needed to offer appropriate treatment [27]. Another major issue is the lack of qualified healthcare workers, which leaves many medical facilities understaffed [22]. The lack of knowledge and instruction on sexual health is another issue. Many young people and their families do not understand the dangers of unprotected sex or the advantages of contraception. Adolescents find it difficult to seek treatment because of the stigma attached to sex, which makes it difficult to execute effective therapies [28].

Recommendations for future research and policy initiatives

To enhance adolescent sexual health services in rural central India, it is imperative to allocate resources for infrastructure and training of healthcare workers, foster community awareness on sexual health, and establish youth-friendly services. Overcoming the challenges of stigma and inadequate knowledge necessitates collaborative efforts involving government, NGOs, and community leaders to create a supportive environment for comprehensive and accessible sexual healthcare [29]. Policymakers and healthcare providers should concentrate on expanding access to sexual health education and services to address adolescent sexual behavior in rural central India [26]. Adolescents should get high-quality treatment; thus, policies should be put in place to enhance financing for medical facilities and train medical staff. To increase knowledge of the significance of safe sex practices, the government should also concentrate on boosting sexual health education in schools and communities [30]. It is necessary to research to determine what influences teenagers' risky sexual behavior in rural central India. To encourage safe sexual behavior, the findings should be used in the creation of evidence-based policies and programs. The social and cultural norms that support stigma and prevent the promotion of safe sex practices should also be addressed with strategies [31].

Preventive measures and proposed recommendations to promote safe sexual behavior

Adolescents in rural central India might be encouraged to engage in safe sexual behavior through a variety of interventions and preventative measures. These consist of (a) providing access to sexual health education and counseling services in schools and health centers [12,32], (b) providing access to contraception and other family planning services, (c) promoting condom use and safe sex practices, (d) encouraging parents and families to talk openly about sex with their children [11], and (e) raising awareness about the risks associated with unprotected sex and the importance of practicing safe sex [33].

Limitations

The limitation of this study includes existing literature, potentially affecting data precision and generalization challenges due to the lack of specificity to particular regions or communities. While government and NGO initiatives are mentioned, their effectiveness is not thoroughly evaluated.

## Conclusions

The challenging issue of teenage sexual behavior in rural central India requires a diversified approach. Promoting safe sexual behavior, enhancing accessibility to sexual health education and services, and addressing social and cultural norms that uphold stigma are all part of this. Teenage sexual behavior is also significantly influenced by gender and socioeconomic background, with females and low-income homes being more vulnerable. A multidimensional approach that takes both institutional and human factors into account, such as increasing access to economic opportunities, education, and gender equity, is required to address this issue. Adolescents in rural areas in central India can access the knowledge, tools, and support they need to decide what is best for sexual health and well-being by addressing these aspects.
